# Strategy for Generating Giant Unilamellar Vesicles with Tunable Size Using the Modified cDICE Method

**DOI:** 10.1021/acssynbio.5c00026

**Published:** 2025-06-19

**Authors:** Ariel Chen, Shachar Gat, Lior Ohana, Evgenee Yekymov, Yoav Tsori, Anne Bernheim-Groswasser

**Affiliations:** † Department of Chemical Engineering, 26732 Ben-Gurion University of the Negev, Beer-Sheva 84105, Israel; ‡ Ilse Kats Institute for Nanoscale Science and Technology, Ben Gurion University of the Negev, Beer-Sheva 84105, Israel

**Keywords:** GUVs, modified cDICE method, size selection, encapsulation efficiency, total GUV yield, emulsion-based GUV production

## Abstract

Our study investigates an optimization strategy that uses a size cutoff in giant unilamellar vesicles (GUVs) generated from water-in-oil (W/O) emulsion droplets using the modified continuous droplet interface crossing encapsulation method. While this method is rapid and cost-effective and yields high encapsulation efficiency, it suffers from a broad, poorly controlled size distribution of vesicles, a significant drawback for the construction of artificial cells. We address this by systematically varying key parameters, such as chamber rotation time, angular frequency, and inner solution density, to refine the GUV size distribution. Our study highlights the importance of these parameters as practical experimental knobs for the refinement of GUVs size. Our results are supported by a physical model, which helps explain the observed size selection phenomena. We also examine how the salinity of the inner solution affects the encapsulation efficiency, finding that a high efficiency is maintained even at physiologically relevant salt concentrations. Our approach offers a practical method for selecting vesicle sizes, thereby facilitating the creation of cell-sized compartments with biologically relevant properties for synthetic biology applications.

## Graphical abstract

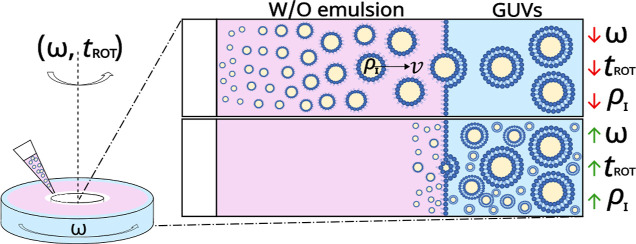

Synthetic cells are invaluable tools for exploring the complexities of cellular functions and advancing the design of novel biotechnologies. By constructing artificial cells, scientists can investigate fundamental biological processes in a simplified and controlled environment, leading to new insights and innovations. Various approaches exist for producing cell-mimicking compartments, including water in oil droplets,^1^ polymersomes,^2,3^ and liposomes.^3–5^

Giant unilamellar vesicles (GUVs) stand out for several reasons. Like living cells, GUVs are large spherical compartments enclosed by a lipid bilayer. Their cell-sized dimensions (5–50 μm) that surpass the optical resolution limit facilitate their investigation by using light microscopy. Their structural similarity to real cells, in terms of size and lipid composition, coupled with their customized nature, makes GUVs ideal for using them for probing cellular processes under controlled conditions and for developing bioinspired applications in medicine, diagnostics, and synthetic biology.

GUVs serve as a versatile model system for characterizing the mechanical properties of lipid membranes, with or without embedded membrane proteins.^6–10^ GUVs are also useful for studying the impact of membrane-bound proteins and membrane curvature on the self-organization of cytoskeletal networks^11,12^ and the effect of integrin-mediated surface adhesion on cell spreading.^13^ Additionally, vesicles serve as bioreactors,^14^ which provides a controlled environment for biochemical reactions and can also be utilized in drug delivery applications.^15^

GUVs can be formed by different methods and technologies (e.g., electroformation,^16^ gel-assisted swelling,^17^ inverted emulsion,^18^ and microfluidic-assisted platforms^1,19^ ). These techniques differ in the yield and encapsulation efficiencies as well as in the size range and polydispersity of the generated GUVs. Some techniques are limited to using lipid compositions with a low proportion of charged lipids and solutions with a relatively low ionic strength such as the electroformation technique. In particular, the use of an electric field limits the use of sensitive biological materials, which are required, notably, to develop a synthetic cell. Many of these methods also involve a time-consuming process to generate vesicles, which limits their use for observing dynamic transient states.

The continuous droplet interface crossing encapsulation (cDICE) method offers several advantages over these techniques. It is a quick and inexpensive method that demonstrates a high yield of GUV production and an elevated encapsulation efficiency.^20^ Furthermore, it can work with lipid compositions containing high proportions of charged lipids and with solutions of high ionic strength such as those present in the cellular environment.^21^

The cDICE method consists of three main steps. The first includes the preparation of a lipid-in-oil mixture (LOM) with mass density ρ_LOM_ and dynamic viscosity μ_LOM_ (Figure 1) through a process called “solvent-shifting” or “Ouzo Effect”.^23^ In this step, lipids are first dissolved in a good solvent (e.g., chloroform) and then mixed with a nonmiscible oil phase (e.g., a mineral and silicon oil mixture^20^), which triggers the formation of lipid aggregates (blue and red spots in the schematic diagram/image (i) in Figure 1). Then, an aqueous phase (denoted as the “outer solution”, O) and the LOM are dripped in sequential order into a chamber rotating at an angular frequency ω (Figure 1). The applied centrifugal forces trigger their phase separation, with the “O” phase being pushed toward the outer chamber edge owing to its higher mass density, ρ_O_, and the establishment of a lipid monolayer at their interface. In the third step, aqueous droplets of the solution (denoted as the “inner solution” I) are dripped into the rotating chamber. Setting the mass density of the different solutions to ρ_I_ > ρ_O_ > ρ_LOM_ ensures that the aqueous droplets are driven toward the LOM/O interface, where they undergo membrane zipping, and are eventually released in the “O” phase as GUVs (see image (ii) in Figure 1).

**Figure 1:**
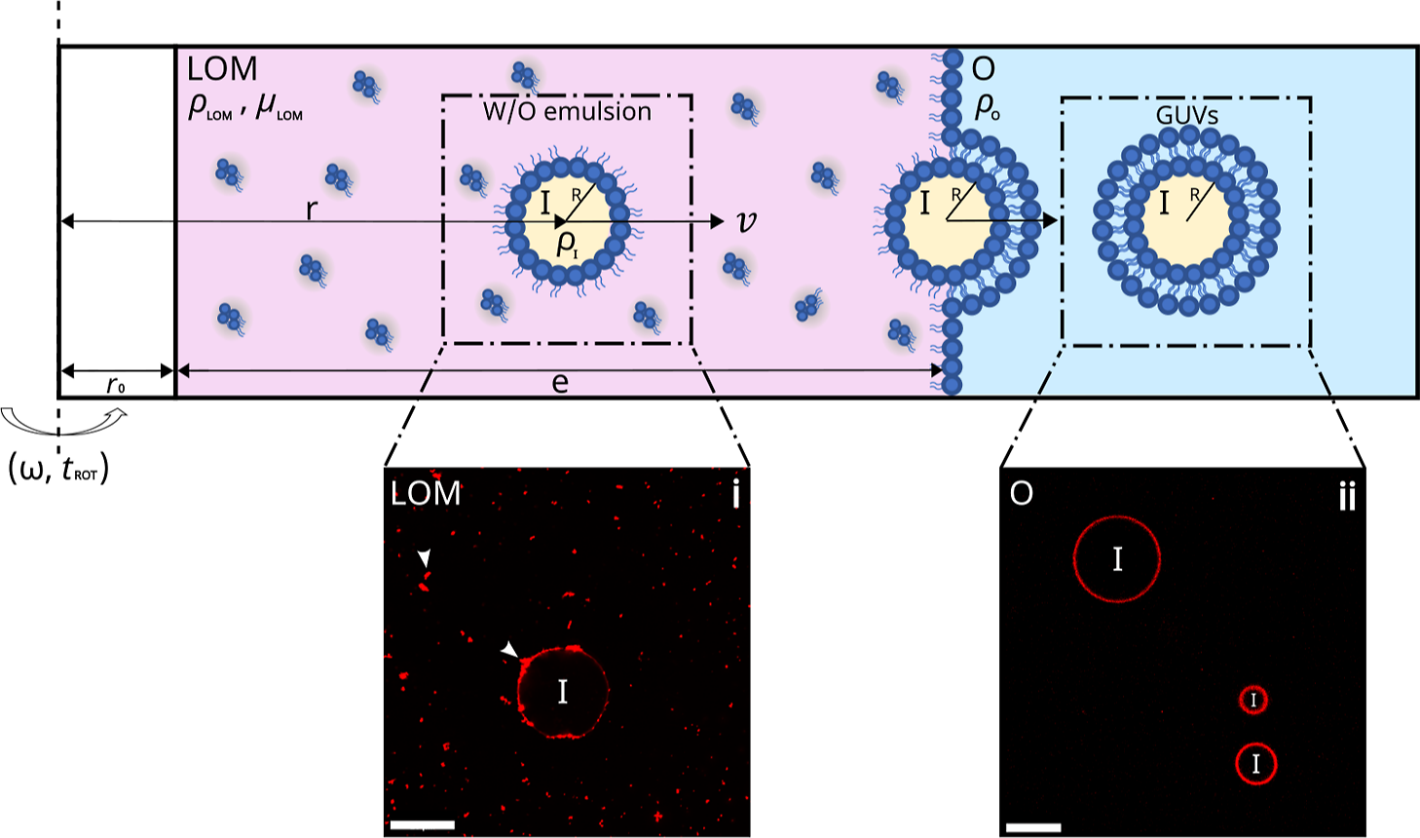
GUVs formation using the modified cDICE method—schematic diagram inspired by ref 22. An outer aqueous phase (“O”) of mass density ρ_O_ and a lipid-in-oil mixture (LOM, lipids are marked in blue) of mass density ρ_LOM_ and dynamic viscosity μ_LOM_ are sequentially dripped into a rotating chamber operating at an angular frequency ω. The centrifugal force generated during rotation induces phase separation and promotes the formation of a lipid monolayer at their interface. Subsequently, water-in-oil emulsion droplets of the sample solution (“I”) and mass density ρ_I_ are introduced in the rotating chamber. These droplets migrate at a velocity *v*, which depends on their radius *R* toward the LOM/O interface. There, they undergo membrane zipping and are released as GUVs in the outer aqueous “O” solution. Only droplets that reach the LOM/O interface at *r*_0_ + *e* in a time shorter than the overall experiment time (i.e., the rotation time *t*_ROT_) end up as GUVs. Also depicted are confocal images of (i) a water-in-oil droplet in the LOM mixture. The red spots are lipid aggregates (white arrow heads) present in the LOM mixture and the W/O droplet interface. (ii) GUVs immersed in the outer “O” phase. Membrane composition (molar percentage): 99.89% DOPC, 0.10% Liss Rhodamine-PE, and 0.01% DSPE-PEG(2000)-Biotin. Scale bars are 20 μm.

Droplets can be introduced bare (i.e., uncoated) using a glass capillary (as per the original cDICE method^21,22,24^ ) or precoated with a lipid monolayer as a water-in-oil (W/O) emulsion (as per the modified cDICE method^20^). Figure 1 (image (i)) depicts an example of such an emulsion droplet. The use of a preformed emulsion is greatly advantageous over the use of naked droplets. First, it is technically less challenging and does not require the use of expensive equipment such as a pipet puller apparatus, except for standard laboratory equipment. Moreover, a key factor to consider is the “time-of-flight”—the time it takes for the droplets to travel through the LOM layer (of thickness *e*) and reach the LOM/O interface. This duration must be long enough to ensure that droplets of all sizes (radius *R*) become fully saturated with lipids, which is a prerequisite for their eventual development into stable GUVs.^24^ The use of W/O lipid droplets minimizes the risk that droplets arriving at the LOM/O interface fail to form stable GUVs. In contrast, with naked droplets, only about 40% convert into GUVs (defined by the authors as the GUVs’ production yield), while the remaining droplets are released into the outer solution.^24^

However, a major limitation of the modified cDICE method is its difficulty in consistently controlling the size of the generated GUV.^20^ This inconsistency arises because the GUV size distribution is determined by the initial droplet size distribution. Unlike the conventional cDICE method, which uses a glass capillary with a diameter of a few to tens of micrometers, thereby constraining the size of the introduced naked droplets and resulting GUVs (typically 3–66 μm depending on the capillary diameter),^24^ the modified approach relies on W/O droplets whose sizes are uncontrolled and can vary widely.^20^

Here, we propose an optimization strategy that allows for a size cutoff in vesicles generated from W/O emulsion droplets and the conditions for achieving effective size selection. We demonstrate how such a size selection can be achieved through proper adjustments of system parameters, such as the chamber rotation time (*t*_ROT_) and angular frequency (ω), or the inner solution density (ρ_I_). Our experimental results are supported by a physical model, which helps uncover the mechanism behind the observed size selection effects and also link key experimental parameters to GUV characteristics.

## Results and Discussion

### In Situ Size Selection of GUVs

#### Effect of Rotation Time (*t*_ROT_)

GUVs were generated using the modified cDICE method.^20^ We conducted a set of experiments at a fixed rotation speed ω = 1600 rpm and an inner solution density ρ_I_ = 1040 kg/m^3^ to explore the effect of rotation time *t*_ROT_ on the GUV size distribution (Figure 2). The experiments include the following time steps from which we calculate the rotation time: (i) the emulsion dripping time = 40 s, constant in all studied systems, (ii) the time the chamber rotates steadily at ω = 1600 rpm—four different times were tested: 15, 45, 180, and 1200 s, and (iii) the time it takes for the chamber to decelerate from ω = 1600 to 0 rpm at a rate of 100 cycles/s (= 16 s). In Figure 2, we show the results obtained for the corresponding four rotation times: 71, 101, 236, and 1256 s.

**Figure 2:**
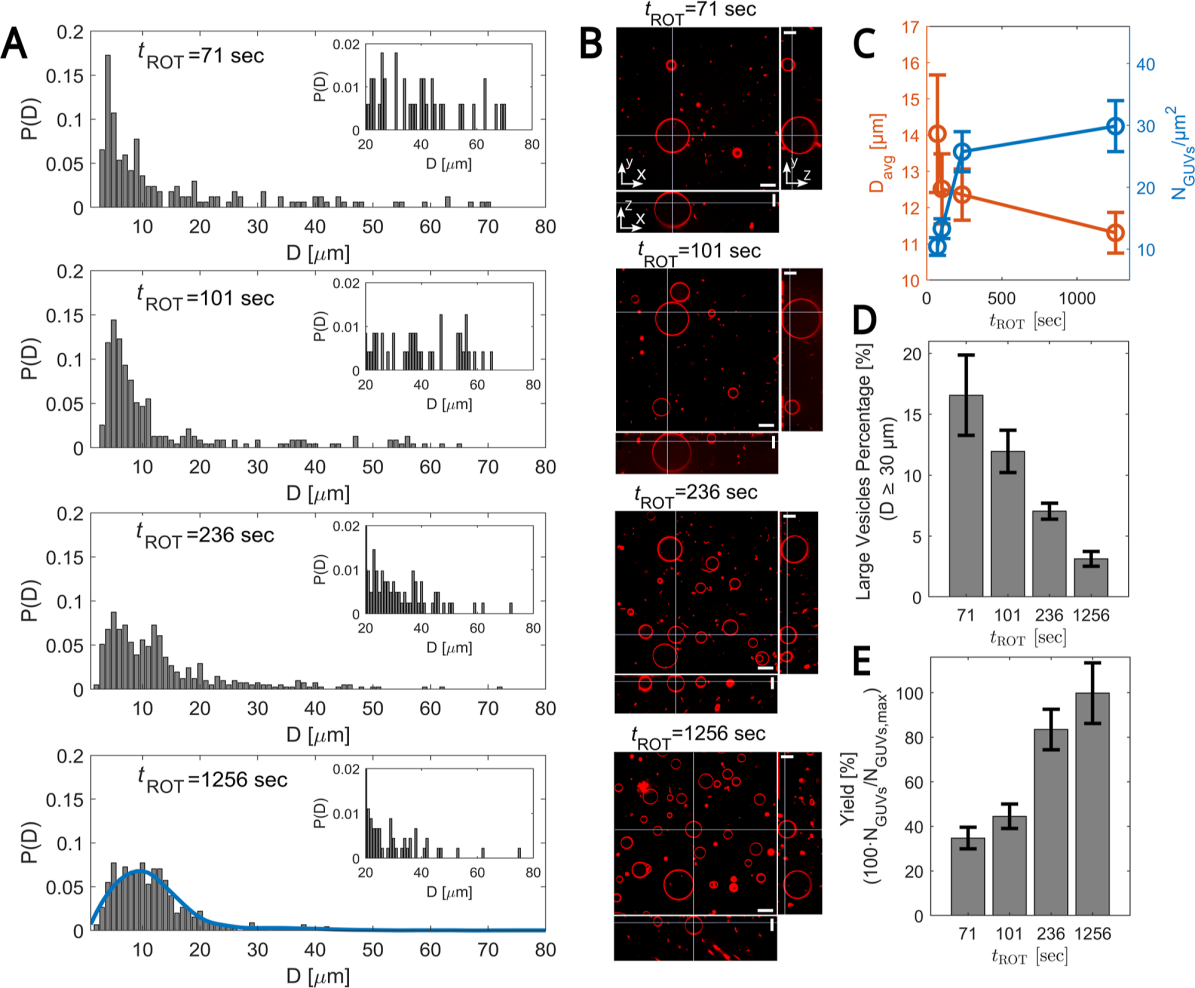
Effect of rotation time (*t*_ROT_) on GUVs’ size distribution. (A) The panels show the probability distribution function *P*(*D*) for vesicle diameter *D* at a fixed angular frequency ω = 1600 rpm and inner solution density ρ_I_ = 1040 kg/m^3^ for four different rotation times = 71, 101, 236, and 1256 s (top to bottom panels). Using quadratic regression, the blue curve fits to the base distribution, as measured from the longest experiment (*t*_ROT_ = 1256 s). Insets: Zoom-in of the *P*(*D*) for *D* values ≥ 20 μm. (B) Spinning disk confocal images of GUVs in the *xy* cross-section (top view), the *xz* cross-section (bottom side view), and the *yz* cross-section (right side view) for the same running conditions as in (A). Images were corrected for index refraction mismatch between the objective immersion liquid and our aqueous sample.^25,26^ Scale bars are 20 μm. Membrane composition (molar percentage): 99.89% DOPC, 0.10% Liss Rhodamine-PE, and 0.01% DSPE-PEG(2000)-Biotin. (C) GUVs diameter *D*_avg_ (orange) and GUVs density (number per unit area) (blue) vs *t*_ROT_. (D) Percentage of large vesicles (*D* ≥ 30 μm) vs *t*_ROT_. (E) GUVs yield vs *t*_ROT_. For each experiment, the yield is calculated as the ratio of the number of vesicles obtained at a specific rotation time to the maximum average number of GUVs, *N*_GUVs,max_ = 510, from three independent experiments, obtained at the longest rotation time (*t*_ROT_ = 1256 s). (C–E) Values are averaged over three independent experiments. The total number of GUVs used for data analysis: 382, 498, 921, and 1085 for *t*_ROT_ = 71, 101, 236, and 1256 s, respectively. Error bars indicate the standard deviation of experimental values.

The histograms of the probability distribution *P*(*D*) for GUV diameters *D* at different *t*_ROT_ and the corresponding spinning disk confocal images of the generated GUVs for the same running conditions are displayed in Figure 2A,B, respectively. The insets in Figure 2A provide more detailed views of the larger size ranges. Our data show that as the rotation time decreases, the distribution shifts toward higher GUV diameters, with fewer observed GUVs (Figure 2B,C) but a higher frequency of larger ones (Figure 2A).

These results are further supported by Figure 2D which shows that the percentage of larger GUVs (*D* ≥ 30 μm) is highest at the shortest rotation time of 71 s and decreases monotonically as the rotation time increases. However, this increase in large GUVs is accompanied by a reduction in overall GUV yield, defined as the percentage of GUVs obtained at a specific rotation time, *N*_GUV_, compared to the maximal number of vesicles, *N*_GUV,max_, which is obtained at the longest rotation time (*t*_ROT_ = 1256 s), highlighting a trade-off between selecting larger GUVs and production efficiency (Figure 2E). This shift in distribution supports the idea that shorter rotation times favor the arrival of large W/O droplets to the LOM/O interface. This size sorting effect leads to the observed increase in average diameter with decreasing rotation time, shown in Figure 2C. However, this size sorting is negligible, as the GUVs distribution does not change with a further increase in rotation time (not shown).

As such, the distribution measured at long rotation time (*t*_ROT_ = 1256 s) provides a direct and quantitative measure of the W/O droplet distribution introduced in the system, which we use as input for the theoretical model (see Section Theoretical Model).

#### Effect of Angular Frequency (ω)

We now turn to explore the effect of varying the rotation frequency ω for a rotation time of 45 s and the inner solution density ρ_I_ = 1040 kg/m^3^ on the GUV size distribution. In Figure 3, we show the data obtained for ω = 1200, 1600, and 2000 rpm and for the corresponding rotation times *t*_ROT_ of 97, 101, and 105 s (including the 40 s dripping time and the ω-dependent deceleration time). As in the effects of varying *t*_ROT_, we find that as ω decreases, the distribution shifts toward higher GUV diameters, accompanied by improved size sorting (Figure 3A), with fewer observed GUVs (Figure 3B,C) but a higher frequency of larger ones (Figure 3A). It is also accompanied by a reduction in the total yield (Figure 3E), calculated here relative to the number of GUVs obtained at the highest angular frequency (2000 rpm), consistent with the results observed for the effect of the rotation time. These results are further supported by Figure 3D, showing that the percentage of larger GUVs (*D* ≥ 30 μm) is highest at the lowest ω = 1200 rpm and decreases monotonically as the rotation speed increases. As a result, the mean GUV diameter also increased (Figure 3C).

**Figure 3:**
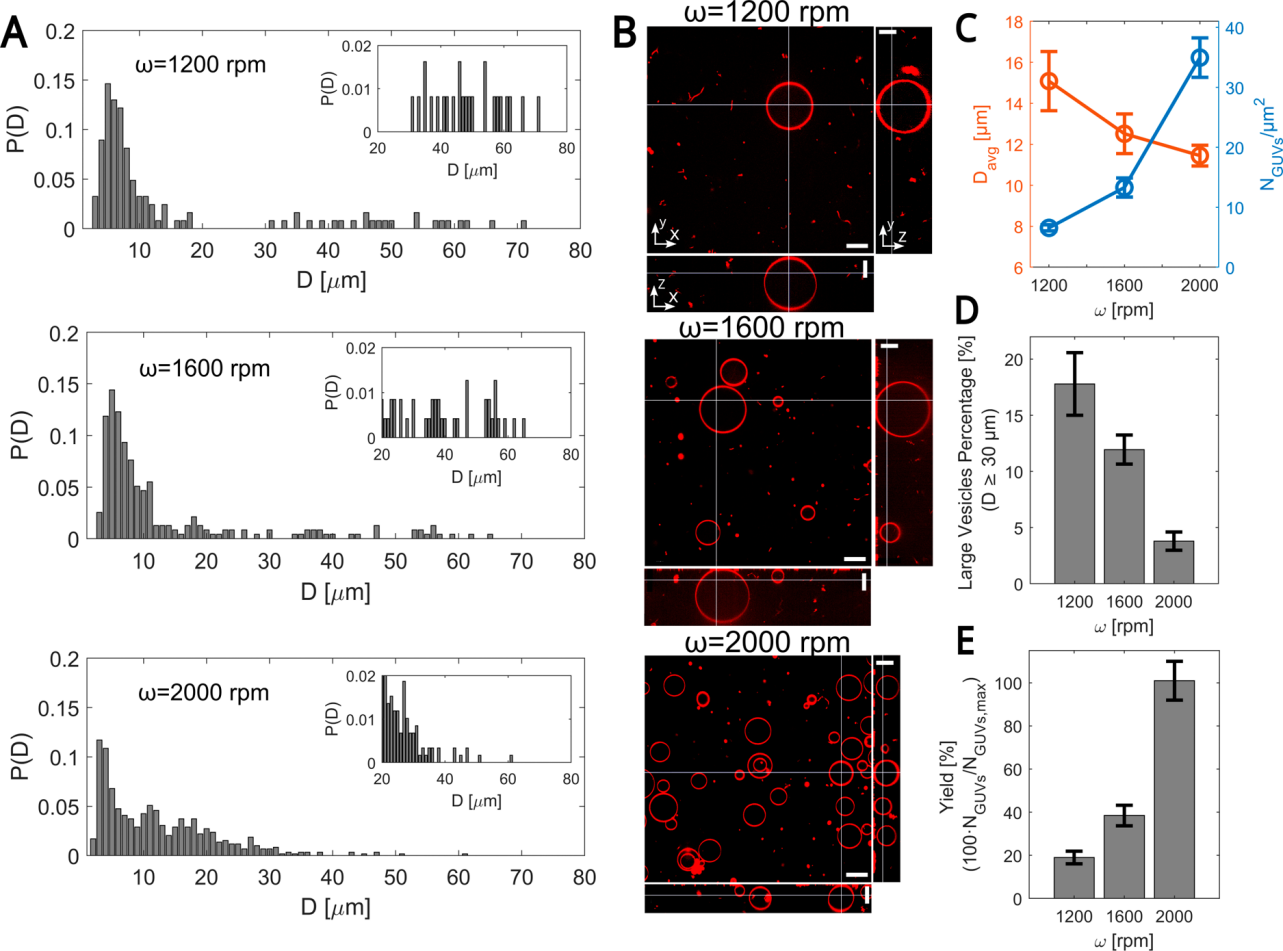
Effect of the angular frequency (ω) on GUVs’ size distribution. (A) The panels show the probability distribution function *P*(*D*) for vesicle diameter *D* at a fixed inner solution density ρ_I_ = 1040 kg/m^3^ for variable ω values of 1200, 1600, and 2000 rpm (top to bottom). The rotation time *t*_ROT_ is 40 + 45 = 85 s plus the deceleration time, yielding: 97, 101, and 105 s, respectively. Insets: Zoom-in of *P*(*D*) for *D* ≥ 20 μm. (B) Spinning disk confocal micrographs of GUVs in the *xy* cross-section (top view), the *xz* cross-section (bottom side view), and the *yz* cross-section (right side view) for the same running conditions as in (A). Images were corrected for the index of refraction mismatch between the objective immersion liquid and our aqueous sample.^25,26^ Scale bars are 20 μm. Membrane composition as in Figure 2B. (C) GUVs diameter *D*_avg_ (orange) and GUVs density (blue) vs ω. (D) Percentage of large vesicles (*D* ≥ 30 μm) vs ω. (E) GUVs yield vs ω. For each experiment, the yield is calculated as the ratio of the number of vesicles obtained at a specific angular frequency to the maximum average number of GUVs, *N*_GUVs,max_ = 590 (average value from three independent experiments), obtained at the highest angular frequency (ω = 2000 rpm). (C–E) Values are averaged over three independent experiments. The total number of GUVs used for data analysis: 248, 498, and 1291 for ω = 1200, 1600, and 2000 rpm, respectively. Error bars indicate the standard deviation of experimental values.

#### Effect of Inner Solution Density (ρ_I_)

We carried out experiments at a fixed rotation speed ω = 1200 rpm and a time *t*_ROT_ of 67 s to investigate how the inner solution density influences the size distribution and overall yield of the GUVs (see Figure 4). To vary ρ_I_, we adjusted the volume percentage of Optiprep, an inert solute, from 0% to 30%, which changed the density from 1020 to 1110 kg/m^3^. Furthermore, to evaluate the encapsulation efficiency and its dependence on ρ_I_, 13 μM fluorescein isothiocyanate (FITC) isomer I was added to the inner solution (see the caption of Figure 4 for details).

**Figure 4:**
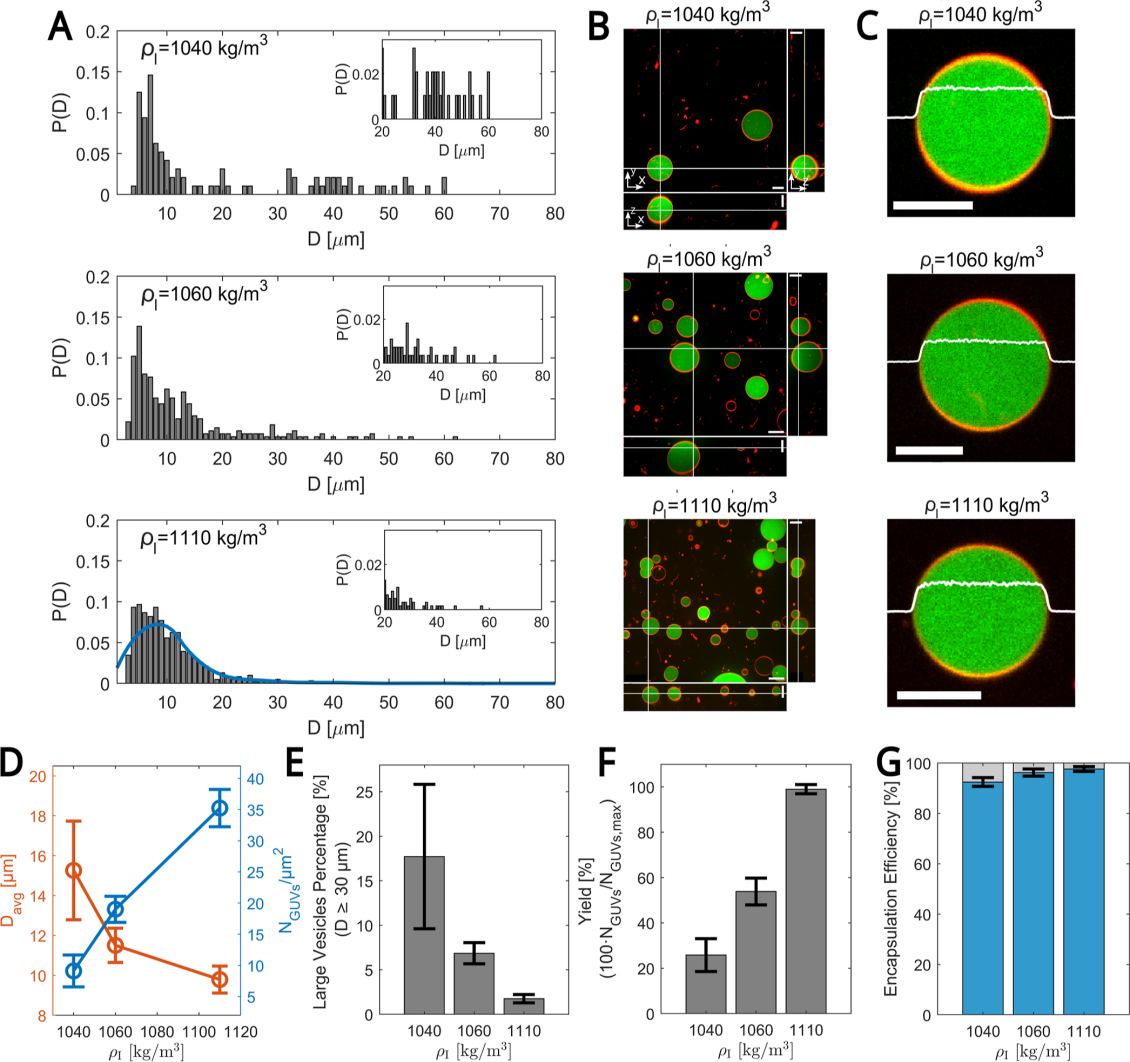
(A) Probability distribution function *P*(*D*) for vesicle diameter *D* at a fixed angular frequency ω = 1200 rpm and rotation time *t*_ROT_ = 40 + 15 + 12 = 67 s for three different inner solution densities ρ_I_ = 1040 kg/m^3^, 1060 kg/m^3^, and 1110 kg/m^3^ (top to bottom panels). The blue curve at the highest inner solution density (ρ_I_ = 1110 kg/m^3^) is a quadratic regression fit. Insets: Zoom-in of *P*(*D*) for *D* ≥ 20 μm. (B) Spinning disk confocal images of GUVs in the *xy* cross-section (top view), the *xz* cross-section (bottom side view), and the *yz* cross-section (right side view) for the same running conditions as in (A). Images were corrected for the index of refraction mismatch between the objective immersion liquid and our aqueous sample.^25,26^ Scale bars are 20 μm. Membrane composition (as in Figure 2B). Inner solution composition: 13 μM FITC, 200 mM Glucose, 10 mM Tris-HCl (pH = 7.4), and 6.5–30.0 vol % Optiprep. (C) Zoom-in on a representative GUV for each inner solution density condition. The white line indicates the intensity line scan. Scale bars are 10 μm. Membrane and inner solution compositions are as in (B). (D) GUVs diameter *D*_avg_ (orange) and GUVs density (blue) vs ρ_I_. (E) Percentage of large vesicles (*D* ≥ 30 μm) vs ρ_I_. (F) GUVs yield vs ρ_I_. For each experiment, the yield is calculated as the ratio of the number of vesicles obtained at a specific inner solution density to the maximum average number of GUVs, *N*_GUVs,max_ = 600 (average value from three independent experiments), obtained at the highest inner solution density (ρ_I_ = 1110 kg/m^3^). (G) Encapsulation efficiency vs ρ_I_. (D–G) Values are averaged over three independent experiments. The total number of GUVs used for data analysis: 313, 683, and 1301 for ρ_I_ = 1040, 1060, and 1110 kg/m^3^, respectively. Error bars indicate the standard deviation of experimental values.

Figure 4 presents the results for the highest densities: 1040, 1060, and 1110 kg/m^3^. For 1020 kg/m^3^, no GUV were formed (not shown). Similarly to the effects observed when varying *t*_ROT_ and ω, a decrease in the inner solution density shifts the size distribution toward the larger GUV diameters, resulting in fewer overall GUVs (Figure 4B,D) but a higher frequency of larger ones (Figure 4A). These results are further supported by Figure 4E, which shows that the percentage of large GUVs (with *D* ≥ 30 μm) is highest at the lowest density (1040 kg/m^3^) and decreases monotonically as ρ_I_ increases. However, this increase in the percentage of large vesicles is accompanied by a reduction in the total GUV yield, calculated relative to the maximum number of GUVs, which is obtained at the largest solution density of 1110 kg/m^3^ (Figure 4F). This shift in size distribution supports the idea that lower solution densities favor the arrival of larger W/O droplets to the LOM/O interface at the expense of smaller ones. Consequently, this size sorting effect results in an increase in the average vesicle diameter as ρ_I_ decreases, as shown in Figure 4D.

The fluorescent dye FITC enabled us to assess the encapsulation efficiency by comparing the fluorescence intensity inside the GUVs with that of the surrounding background (Figure 4C). We defined successful encapsulation as a condition in which the fluorescence within GUVs exceeds the average background by four standard deviations (SD) (see Figure S1 for details). Under all conditions tested, the encapsulation efficiency remained very high (over 92%) and was independent of the density of the inner solution (Figures 4G and S1). Furthermore, these encapsulation efficiencies are significantly higher than those achieved with the original cDICE method, where only 40% of the naked droplets transformed into GUVs.^24^ This further indicates that the use of W/O emulsion droplets significantly promotes the formation of mechanically stable GUVs, which is not the case for naked droplets.

Finally, we observe that the distribution at the highest inner solution density (Figure 4A, 1110 kg/m^3^) closely resembles the distribution achieved with a long rotation time (Figure 2A, *t*_ROT_ = 1256 s). This similarity indicates that, like the long rotation time, the distribution at the highest density directly and quantitatively reflects the W/O droplet distribution introduced in the system. It also suggests that increasing the inner solution density can reduce the rotation time needed to obtain similar GUV characteristics.

### Theoretical Model

A model was developed to clarify the described phenomena by analyzing the balance of forces acting on a moving aqueous droplet within the cDICE chamber. The parameters used in this model are summarized in Table 1. The Weber number *We* quantifies the ratio between the stress exerted on the droplet by the surrounding oil phase, leading to distortion from a spherical shape, to the stress by surface tension, tending to preserve a perfectly spherical shape^27,28^

1 $$We=\frac{\rho_{LOM}v^{2}D}{\sigma }$$

Here, *v* is the velocity of the droplet in the radial direction relative to the embedding medium and *D* is the droplet’s diameter. As a crude estimate, we use ρ_LOM_ ≈ 1000 kg/m^3^, σ ≈ 50 mN/m, *D* ≈ 20 μm, and *v* ≈ 1 mm/s, and find that *We* ∼ 10^–6^. Since *We* ≪ 1, we conclude that droplets remain nearly spherical.^29,30^

**Table 1:** Parameters Description

parameter	description
μ_LOM_	oil mixture viscosity
ρ_LOM_	oil mixture density
ρ_O_	outer solution density
ρ_I_	inner solution density
υ	droplet velocity
*R*	droplet radius
*r*	travel distance
*r*_0_	distance of the oil layer from the rotation axis
*e*	oil layer thickness
ω	angular frequency
*t*_ROT_	rotation time

The acceleration r̈ for a spherical droplet of radius *R* is given by

2 $$\frac{4\pi }{3}R^{3}\rho_{I}\mathrm{r̈}=\frac{4\pi }{3}R^{3}\Delta \rho \omega^{2}r-6\pi \mu_{LOM}Rṙ$$

The first term on the right-hand side is the centrifugal force, while the second term is the drag (Stokes) force acting on it.

The solution to this second-order linear ordinary equation is

3 $$r\left(t\right)=c_{1}e^{\alpha_{1}t}+c_{2}e^{\alpha_{2}t}$$

where the time constants α_*i*_ (*i* = 1, 2) are the roots of

4 $$\alpha_{i}^{2}+\frac{9\mu_{LOM}}{2R^{2}\rho_{I}}\alpha_{i}-\frac{\Delta \rho }{\rho_{I}}\omega^{2}=0$$

The constants *c*_1_ and *c*_2_ are fully determined from the initial conditions *r*(*t* = 0) = *r*_0_ and ṙ ­(*t* = 0) = 0, which translate to the two linear equations *c*_1_ + *c*_2_ = *r*_0_ and α_1_ *c*_1_ + α_2_ *c*_2_ = 0. The system has two inherent time scales: *T*_1_ = 2*R* ^2^ρ_I_/9μ_LOM_ and $T_{2}={\left(\omega \sqrt{\Delta \rho /\rho_{I}}\right)}^{-1}$ . In the values of parameters involved, *T*_2_ ≫ *T*_1_ meaning the left-hand side in eq 2 nearly vanishes, as originally proposed,^24^ in agreement with the small Reynolds number *Re* ≈ 10^–3^ (viscous drag forces dominate the droplets’ motion).

Figure 5 shows the theoretical predictions for the three droplets with different radii. The smallest droplet (diameter 12 μm) travels slower than the other two (diameters 16 and 20 μm). This is because the ratio of volume forces (∼*R* ^3^) to surface forces (∼*R*) is lower than that for larger droplets. In all cases, the trajectories *r*(*t*) are almost linear over short times. In the simulation, we used a constant value of ω for a certain time followed by a linearly decreasing ω­(*t*) as prescribed by the experimental protocol (see Section Materials and Methods for details). The curves *r*(*t*) level off at the end due to the deceleration period. Within the 97 s total rotation time droplets with diameters of 16 and 20 μm traverse the entire LOM, while the smallest droplet does not. For example, after 40 s, only the largest droplet (*D* = 20 μm) reaches the interface and transforms into a vesicle.

**Figure 5:**
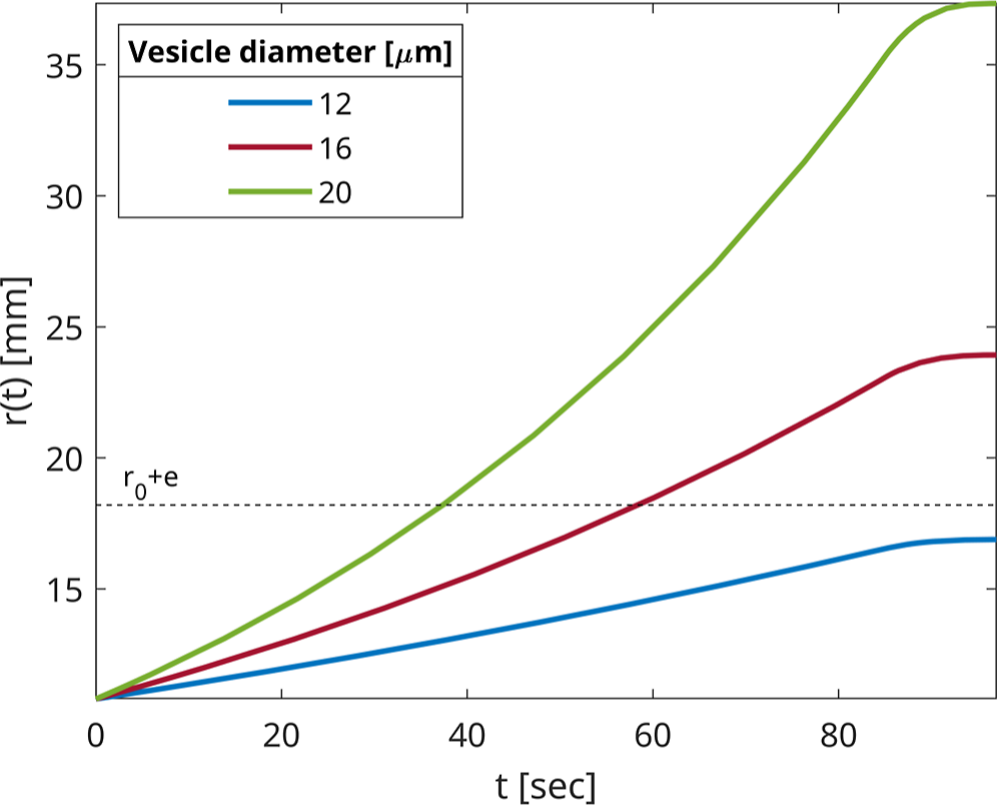
Three trajectories *r*(*t*) found from eq 2 for three droplet diameters at a rotation time of 97 s. We used ω = 1200 rpm for a rotation time of 85 s and ω linearly decreasing to zero at a rate of 100 cycles/s in the final 12 s. The horizontal dashed line is the outer radius, *r* = *r*_0_ + *e*. In this and in other model results, we used *r*_0_ = 10.8 mm, *e* = 7.4 mm, μ_LOM_ = 0.00564 Pa s, ρ_I_ = 1040 kg/m^3^, and ρ_LOM_ = 898 kg/m^3^.

One may also look at the effect of varying the rotation time *t*_ROT_ at a fixed rotation speed ω = 1600 rpm. In Figure 6A–C, we calculated the GUV size distribution *P*(*D*), defined as the relative probability of finding a droplet of diameter *D*. In Figure 6A, there are four panels for four times: 71, 101, 236, and 1256 s. In each panel, the blue curve is a fit to base distribution *P*(*D*) in the absence of rotation, taken from the experiments with the longest time of 1256 s. The dashed vertical lines mark the value of droplet diameter below which droplets cannot cross the LOM at the time of the experiment. The shaded region marks the droplet sizes that cross the LOM/O interface. The red curve is the predicted biased distribution of droplets *P*′(*D*) reaching the outer radius, located *r*_0_ + *e*, at time *t*_ROT_ (Section Materials and Methods). It is clear that the shorter the experiment is, the more *P*′(*D*) deviates from the base distribution *P*(*D*). Panels B and C display how the average diameter and the percentage of large vesicles (those with *D* ≥ 30 μm) depend on *t*_ROT_. Both the average vesicle size and the fraction of large vesicles decrease as the rotation time increases, which aligns with our theoretical predictions and experimental observations.

**Figure 6:**
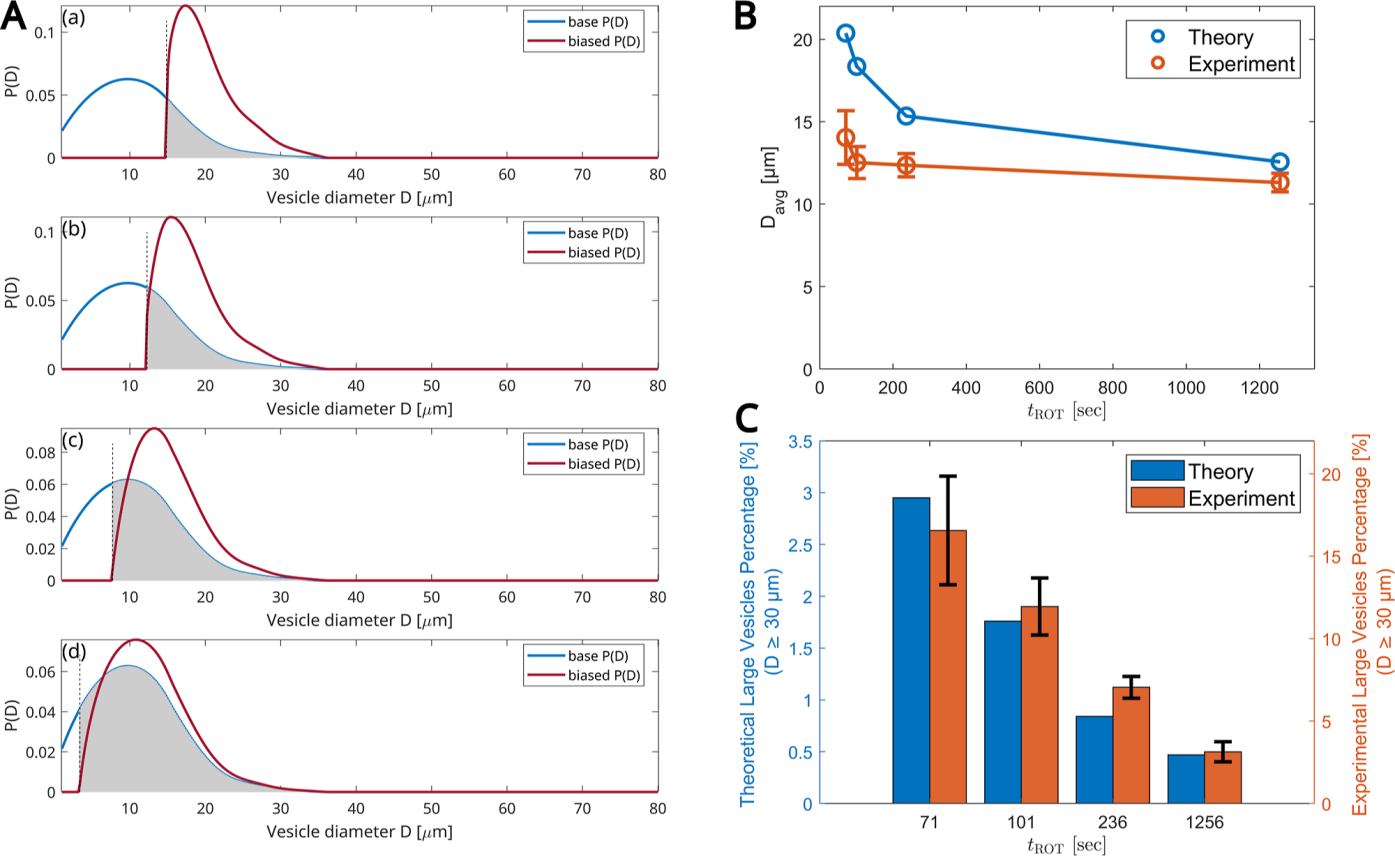
Effects of the rotation time—theoretical model predictions. (A) Panels show the probability distribution function *P*(*D*) for vesicle size *D* at a fixed angular frequency ω = 1600 rpm for four different rotation times *t*_ROT_ = 71, 101, 236, and 1256 s (top to bottom panels). The blue curve is a fit to the base distribution, as measured from the longest experiment (*t*_ROT_ = 1256 s), using quadratic regression. The red curve is the calculated biased *P*′(*D*). (B) Mean diameter vs *t*_ROT_. (C) Percentage of large vesicles (*D* ≥ 30 μm) vs *t*_ROT_. (B,C) Experimental data: values are averaged over three independent experiments. The total number of GUVs used for data analysis: 382, 498, 921, and 1085 for *t*_ROT_ = 71, 101, 236, and 1256 s, respectively. Error bars indicate the standard deviation of experimental values.

In Figure 7A, we simulated experiments with varying values of ω at a given rotation time of 45 s, which corresponds to total rotation times of *t*_ROT_ = 97, 101, and 105 s for the rotation speeds ω = 1200, 1600, and 2000 rpm, respectively. The three panels correspond to the three speeds. In each panel, the blue curve is the base distribution *P*(*D*) in the absence of rotation, taken from the experiment with the longest rotation time of 1256 s. The red curve is the predicted biased distribution *P*′(*D*) of droplets reaching the outer radius, located *r*_0_ + *e*, at a given time *t*_ROT_. The trend of decreasing mean diameter with ω is the same in both theory and experiment (Figure 7B). Similarly, as ω increases, the percentage of large vesicles (with *D* ≥ 30 μm) decreases (Figure 7C).

**Figure 7:**
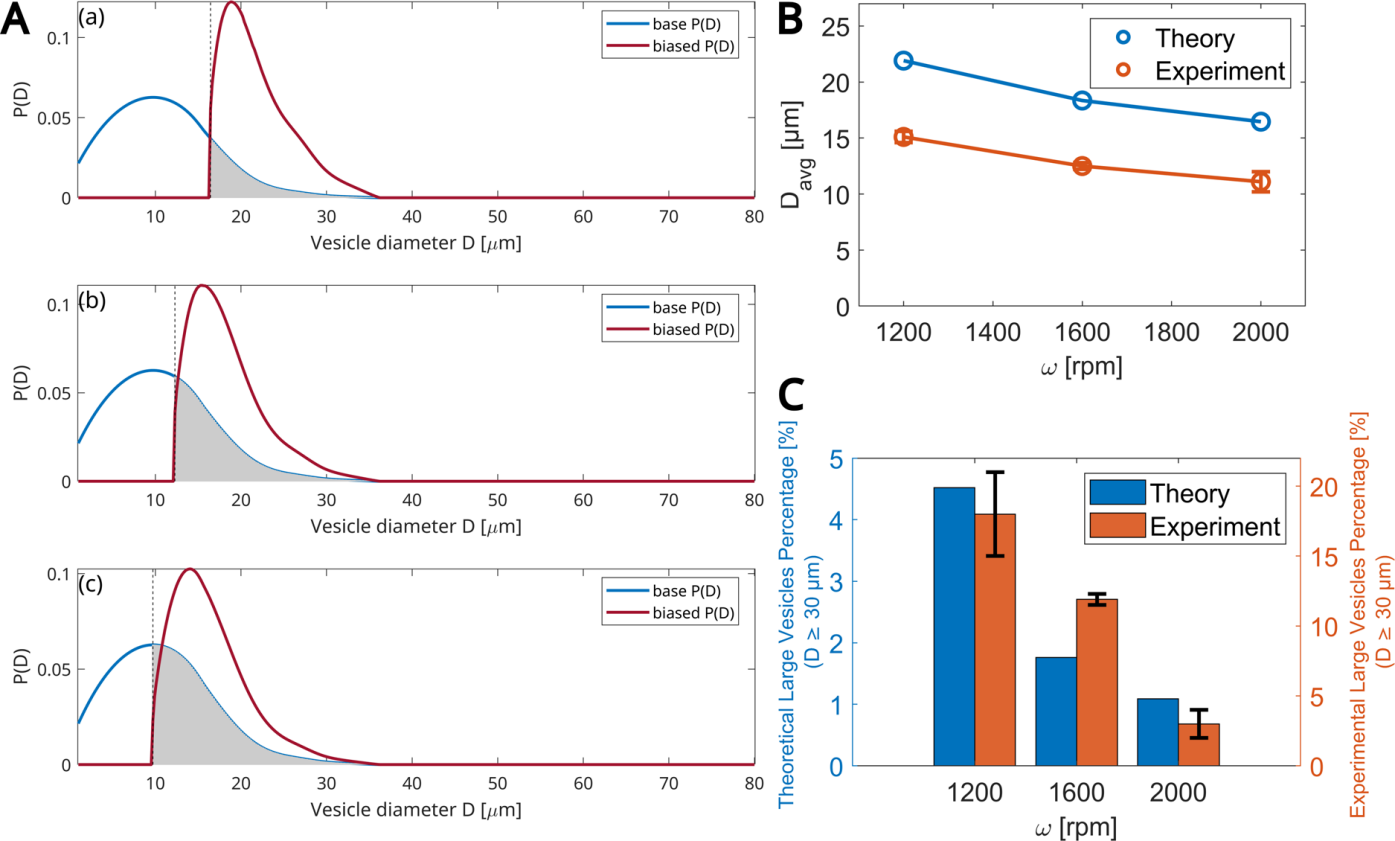
Effects of the angular velocity—theoretical model predictions. (A) ω varies between 1200, 1600, and 2000 rpm (top to bottom panels). The total rotation time is 40 + 45 = 85 s plus the deceleration time. (B) Mean diameter size vs ω. (C) Percentage of large vesicles (*D* ≥ 30 μm) vs ω. (B,C) Experimental data: values are averaged over three independent experiments. The total number of GUVs used for data analysis: 248, 498, and 1291 for ω = 1200, 1600, and 2000 rpm, respectively. Error bars indicate the standard deviation of experimental values.

In Figure 8, we simulate experiments with varying values of ρ_I_ at a given angular velocity and rotation time as used in the experiments (Figure 4). The three panels in A show the results obtained for three solution densities: ρ_I_ = 1040, 1060, and 1110 kg/m^3^, which correspond to the three panels from top to bottom. In each panel, the blue curve represents the base distribution *P*(*D*) taken from the experiments with the longest time of 1256 s, while the red curve is the predicted biased distribution *P*′(*D*). The decreasing trend in the mean vesicle diameter with an increase in ρ_I_ is consistent between the model and the experimental results (Figure 8B). Similarly, as ρ_I_ increases, the proportion of large vesicles (those larger than *D* ≥ 30 μm) decreases (Figure 8C).

**Figure 8:**
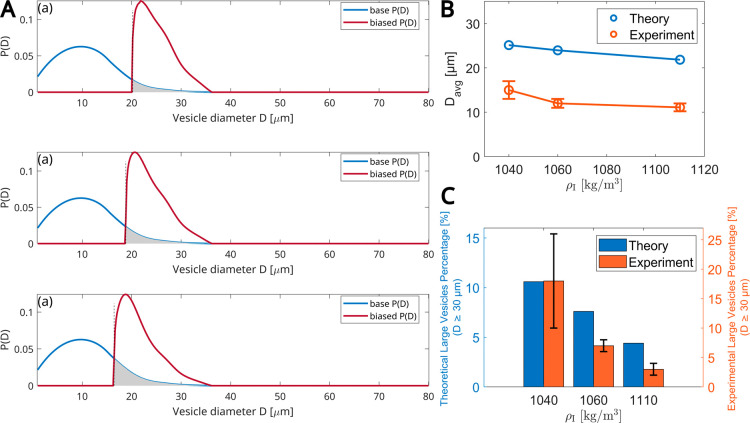
Predictions of the theoretical model for the effect of the inner solution density. (A) ρ_I_ varies between 1040, 1060, and 1110 kg/m^3^ (top to bottom panels) at a fixed angular frequency ω = 1200 rpm. The total rotation time is 40 + 15 + 12 = 67 s. (B) Mean diameter size vs ρ_I_. (C) Percentage of large vesicles (*D* ≥ 30 μm) vs ρ_I_. (B,C) Experimental data: values are averaged over three independent experiments. The total number of GUVs used for data analysis: 313, 683, and 1301 for ρ_I_ = 1040, 1060, and 1110 kg/m^3^, respectively. Error bars indicate the standard deviation of experimental values.

We conclude that the trends observed in both the theoretical model and experimental results consistently show that reducing the angular frequency, rotation time, or inner solution density increases the relative contribution of larger vesicles. This agreement suggests that the model adequately explains the phenomenon. The model also shows that at short rotation times, low angular frequencies, and low solution densities, smaller droplets do not pass to the outer solution and thus do not become vesicles. However, probably due to other mechanisms, such as droplet–droplet interactions, smaller droplets still manage to pass the distance *r*_0_ + *e*, leading to deviations in size distribution and mean diameter values. Despite these differences, the overall trend remains consistent in theory and experiment.

### Effect of Salinity on Encapsulation Efficiency across Systems

After having shown that the encapsulation efficiency of small molecular markers such as FITC remains consistently high (>94%) regardless of solution density (experiments performed in salt-free conditions) (Figures 4 and 9A and S1), our next step consisted of exploring the effect of salinity on encapsulation efficiency.

**Figure 9:**
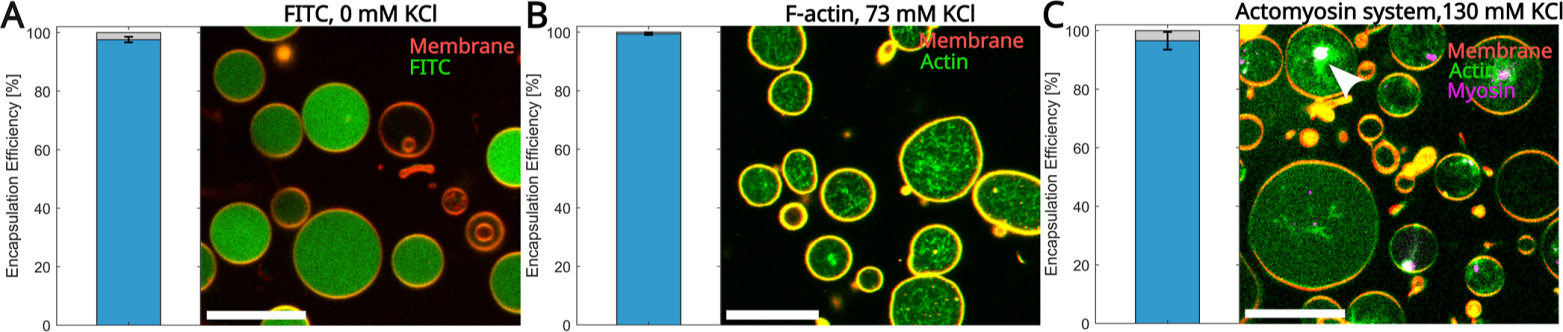
Effect of salinity on encapsulation efficiency in modified cDICE-generated GUVs. (A–C) Left panels present bar plots quantifying the encapsulation efficiency for different salinity conditions and encapsulated systems. Right panels display confocal *xy* cross-section images of the GUVs under different salinity conditions. (A) FITC: 13 μM FITC, 200 mM Glucose, 10 mM Tris-HCl (pH = 7.4), and 30 vol % Optiprep. (B) Preformed actin filaments (F-actin): 1.5 μM G-actin (15 mol % labeled with Alexa-Fluor 488), 2 mM MgCl_2_, 10 mM Tris–HCl (pH = 7.4), 73 mM KCl, 1 mM ATP, 0.2 mM EGTA, and 6.5 vol % Optiprep. (C) Actomyosin system: 2 μM G-actin (15 mol % labeled with Alexa-Fluor 488), 0.1 μM fascin, 0.036 μM myosin II (20% labeled with Alexa Fluor 647) in the form of small filaments each containing 20 myosin dimers,^31^ 2 mM MgCl_2_, 10 mM Tris-HCl (pH = 7.4), 130 mM KCl, 1 mM ATP, 0.2 mM EGTA, and 6.5 vol % Optiprep. The arrowhead in (C) highlights the center of an aster structure. Scale bars are 20 μm. Values are averaged over three independent experiments. The total number of GUVs used for data analysis: 1301, 420, and 274 for FITC, F-actin, and actomyosin system, respectively. Error bars indicate the standard deviation of experimental values.

To assess whether these high encapsulation efficiencies extend to more complicated and biologically relevant systems, we investigated the impact of solution salinity by encapsulating cytoskeletal proteins at physiologically relevant salt concentrations to test how this influences the efficiency of encapsulation in cell-like reconstitution systems. We tested the encapsulation efficiency of (i) preformed actin filaments (F-actin) (Figure 9B) and (ii) of an actomyosin solution, consisting of actin monomers (G-actin), the cross-linker fascin, and myosin II motor proteins (Figure 9C) within the GUVs. The F-actin and actomyosin solutions were encapsulated in the presence of millimolar salt concentrations (73 and 130 mM KCl, respectively).

Analysis revealed that 99.5% of the GUVs include F-actin and about 97% actomyosin networks (Figure S2), indicating a very high encapsulation efficiency, very similar to those measured for FITC under salt-free conditions (Figure S1). Given the relevance of actomyosin systems in synthetic cell models, we also confirmed that myosin motors remained active after encapsulation by showing that they could self-organize actin into structures such as asters and localize in their center (see the arrowhead in Figure 9C) similar to what is observed in experiments performed in bulk solutions.^31,32^

Overall, despite the compositional difference among these three distinct systems (FITC, F-actin, and actomyosin), the encapsulation efficiency remains consistently high. These findings demonstrate the robustness of the modified cDICE method for encapsulating diverse biological systems under different salinity conditions while keeping their inherent activity intact, crucial for various applications in synthetic biology.

## Conclusions

Our study investigates an optimization strategy that uses a size cutoff in vesicles generated from W/O emulsion droplets using the modified cDICE method. Using W/O emulsion droplets, we achieve greater flexibility in adjusting the system parameters, such as the inner solution density, rotation time, and angular speed, without compromising vesicle formation or stability even under conditions of short rotation times, high angular frequencies, or increased inner solution density. Our study highlights the importance of these parameters as practical experimental knobs for the refinement of vesicle size selection while preserving high encapsulation efficiencies and also allowing enrichment of specific vesicle size ranges at the cost of overall yield. This size-based selection strategy is less applicable for naked droplets as many fail to convert into GUVs, leading to unpredictable changes in the initial droplet size distribution.

A potential strategy to further refine the selection of the size of the GUVs could involve reintroducing the GUVs produced back into the chamber for multiple cycles. This iterative process may progressively refine the size distribution, although further experiments are required to verify this effect.

By providing a robust methodology for sorting GUV sizes, our strategy offers a significant advantage to researchers aiming to develop cell-sized compartments with biologically relevant properties for synthetic biology applications.

## Materials and Methods

### Materials

1,2-Dioleoyl-*sn*-glycero-3-phosphocholine (DOPC, 850375C) and 1,2-distearoyl-*sn*-glycero-3-phosphoethanolamine-*N*-[biotinyl­(polyethylene glycol)-2000] (ammonium salt) (DSPE-PEG(2000) Biotin, 880129C) were purchased from Avanti polar lipids in their solubilized form in CHCl_3_. 1,2-Dioleoyl-*sn*-glycero-3-phosphoethanolamine-*N*-(lissamine rhodamine B sulfonyl) (ammonium salt) (Liss Rhodamine PE, 810150P) was purchased from Avanti Polar Lipids as a powder. Density gradient medium (Optiprep, D1556), Silicon oil (317667), Mineral oil (M5904), Casein from bovine milk (C5890), and FITC isomer I (3326-32-7) were purchased from Sigma-Aldrich. d-(+)-Glucose, molecular biology reagent (02194024-CF), EGTA (195174), and Tris-HCl (819620) were purchased from MP Biomedicals. KCl (529552) and MgCl_2_ (442615) were purchased from EMD Millipore Corp. ATP (51963-61-2) was purchased from Roche Life Science Products, and μ-Slide 8 Well high (ibiTreat, 80826) was purchased from IBIDI.

### Methods

#### Protein Purification

G-actin is purified from rabbit skeletal muscle acetone powder by gel filtration,^33^ stored on ice, and used within 3–4 weeks. Actin is labeled on Cys374 with an Alexa-Fluor 488 C5 maleimide (Invitrogen). Myosin II skeletal muscle is purified following ref 34 and labeled with Alexa-Fluor 647 at pairs of engineered cysteine residues.^31,35^ Recombinant GST–fascin was produced following a modified protocol based on the method of ref 36

#### Modified cDICE Method for GUVs Preparation

The various solutions and procedures are based on the protocols described in refs 12, 20, and 22 . All steps are performed at room temperature unless stated otherwise.

#### LOM Preparation

The LOM mixture is prepared following the protocol of ref 20. Briefly, a lipid mixture with molar compositions of 99.89% DOPC, 0.10% Liss Rhodamine-PE, and 0.01% DSPE-PEG(2000) Biotin and a concentration of 6.65 mM were prepared in chloroform and stored under an argon atmosphere at −20 °C until used. In a subsequent step, an oil mixture consisting of 80% silicone oil and 20% mineral oil (vol %) was degassed for 2 h before being added to the lipid mixture to create a 0.417 mM LOM mixture with 6.25 vol % chloroform content. This mixture, which becomes turbid at this lipid concentration,^20,23^ was kept on ice and used within a few minutes.

#### Inner (“I”) and Outer (“O”) Aqueous Solutions

Both solutions include glucose to control their osmolality, which is measured by using an Osmometer (Gonotec Osmomat 3000). The inner solution is supplemented with a variable amount of Optiprep, which can vary between 6.5 and 30 vol % and has an osmolality of 200–350 mosmol/kg, lower by 10 mosmol/kg compared to that of the outer solution.

#### W/O Emulsion Preparation

This step is based on the protocol of ref 20. Briefly, 700 μL of the LOM phase was added to 20 μL of the “I” solution. To facilitate the formation of the W/O droplets, the solution was pipetted nine times with a 1000 μL pipet.

#### Preparation of the GUVs via the Modified cDICE Method

Initially, 700 μL of the “O” solution was introduced in the chamber, which rotates at a constant angular frequency ω (set to 1200, 1600, or 2000 rpm). In a subsequent step, 5 mL of LOM was introduced in the rotating cDICE chamber. The preformed emulsion is then dripped into the chamber (center) using a 1000 μL pipet. This process typically took 40 s. Chamber rotation proceeds at the same constant speed for an additional period of 15, 45, 180, or 1200 s, depending on the experiment, after which ω is gradually decreasing at a rate of 100 cycles/s until halt. At this point, the system consists of two-separated top (LOM) and bottom (GUVs), phases easily detectable when the chamber is tilted by 90°. The chamber is kept in this position for 10 min to promote GUVs’ sedimentation, after which 250 μL of the bottom phase are collected and transferred in a casein-passivated Ibidi well. The wells are passivated with a solution of 2 mg/mL casein in 10 mM Tris-HCl (pH = 7.5), incubated for 15 min, then rinsed first with 250 μL of DDW, and then with “O” solution, and finally they are dried with a flow of N_2_. The same passivation procedure is employed for confocal imaging of the W/O emulsion.

#### LOM Viscosity Measurement

The viscosity of the LOM was determined by a continuous rotation experiment. Measurements were carried out using an MCR 702e MultiDrive rheometer (Anton Paar, Graz, Austria), which is equipped with stainless steel plates with parallel geometry (*d* = 50 mm). The shear rate was conducted between 100 and 500 [1/s], and the viscosity (mPa s) was recorded at 24 °C.

#### Microscopy Technique

Imaging was performed using a Zeiss LSM 880 confocal microscope in AiryScan mode (relevant only for Figure 1) or with a spinning disk confocal microscope equipped with a Yokogawa W1 module and a Prime 95B sCMOS camera (3i, Intelligent Imaging Innovations, Denver, USA). A 63×, 1.4 NA Corr.M27 Oil Immersion Plan-Apochromat objective was used for the imaging. The confocal images were corrected for the index refraction mismatch between the objective immersion liquid and our aqueous sample.^25,26^

#### Data Quantification

The GUVs’ diameter was extracted manually from the 3D confocal images by first identifying for each GUV the *z*-plane where the diameter is the largest. Next, the GUV’s circular shape was fitted to a circle. From these measurements, probability distribution function *P*(*D*) and mean diameter *D*_avg_ were evaluated. Hundreds of vesicles were analyzed for each studied condition (detailed in the figure’s caption). To quantify the number of GUVs that reach the “O” phase per studied condition, we evaluated their number density in units of *N*_GUVs_/μm^2^, averaged over at least eight confocal images (each 210 μm × 210 μm in area). The yield is calculated as the ratio of the number of vesicles obtained at a specific rotation time/angular velocity/inner solution density to the mean maximum number of GUVs, which is obtained at the longest rotation time (1256 s)/highest angular velocity (2000 rpm)/highest inner solution density (1110 kg/m^3^), respectively. The encapsulation efficiency is determined from the fluorescence intensity measured within the GUVs and in the background. All intensity values were normalized to the maximum fluorescence intensity measured in each experiment. Successful encapsulation is considered when the fluorescence signal within a GUV exceeds the average background fluorescence (which is normally distributed) by four SD.

Zen Black 2.1 (Zeiss, Germany), ImageJ, and MATLAB (MathWorks, MA, USA) software were used for data processing, quantification, and analysis.

#### Parameter Values Used for Calculating the Droplets Traveled Distance *r*

All parameter values are estimated at 25 °C. ρ_LOM_ = 900 kg/m^3^, which we estimate from the volume average of the density values of the pure silicone (910 kg/m^3^) and mineral (840 kg/m^3^) oils (Source: Sigma-Aldrich Web site). Similarly, the density of the “I” solution, ρ_I_, was derived from the density values of the various stock solutions used (Optiprep, DDW, Glucose, FITC, and Tris-HCl solutions). μ_LOM_ = 5.64 mPa s is determined experimentally. The cDICE chamber has dimensions of 38 mm in diameter and 7.4 mm in thickness, and combined with the volumes of the LOM (5 mL) and the “O” solution (700 μL), give rise to *r*_0_ = 10.8 mm and *e* = 7.4 mm.

#### Calculation of the Biased Vesicle Size Distribution *P*′(*D*)

*P*′(*D*) was calculated in the following steps. All vesicle diameters *D* were scanned; for a given *D*, the time *t*(*D*) for that particular size to cross the LOM was calculated based on eq 2. The time difference Δ*T*(*D*) was defined for *t*_ROT_ ≥ *t*(*D*) as Δ*T*(*D*) ≡ *t*_ROT_ – *t*(*D*) and 0 otherwise. Δ*T* is proportional to the number of vesicles of size *D* arriving at the outer “O” solution. The biased distribution is then obtained as *P*′(*D*) = *A* × Δ*T*(*D*)*P*(*D*), where the renormalization factor *A* ensures the sum of all probabilities is unity.

## Supplementary Material



## Data Availability

The data that support the findings of this study are available from the corresponding author upon reasonable request.

## Notes

Quantification of the encapsulation efficiency in GUVs for different inner solution densities and salinity (PDF)
