# Supplementary material for: Strategy for Generating Giant Unilamellar Vesicles with Tunable Size Using the Modified cDICE Method
Source: ACS Synth Biol. 2025 Jun 19;14(7):2597–608. doi: 10.1021/acssynbio.5c00026 (PMC12281611; doi:10.1021/acssynbio.5c00026)
Supplement: Supplementary file 1 [file sb5c00026_si_001.pdf]

Strategy for generating Giant Unilamellar Vesicles (GUVs) with tunable size using the modified cDICE method

Ariel Chen [a], Shachar Gat [a], Lior Ohana [a], Evgenee Yekymov [a], Yoav Tsori [a], and Anne Bernheim-Groswasser [a,b] *

[a]Department of Chemical Engineering, Ben-Gurion University of the Negev, Beer-Sheva 84105, Israel.
[b]Ilse Kats Institute for Nanoscale Science and Technology, Ben Gurion University of the Negev, Beer-Sheva 84105, Israel.

*To whom correspondence should be addressed. Email: bernheim@bgu.ac.il

SI Figures

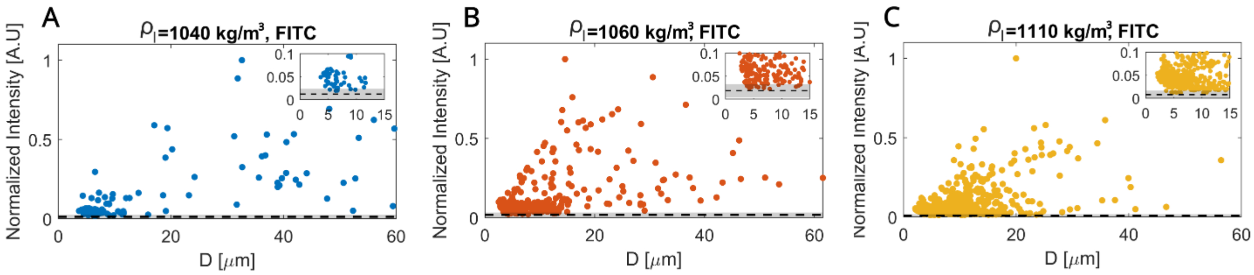

Figure S1: Normalized fluorescence intensity of encapsulated GUVs as a function of vesicle diameter (*D*) for different inner solution densities. Each panel shows the normalized fluorescence intensity (A.U.) of individual GUVs plotted against their diameter (*D*) for inner solution densities of ρ_I= 1040, 1060, and 1110 kg/m³. All intensity values were normalized to the maximum fluorescence intensity measured within each experiment. The dashed lines indicate the mean background intensity, and the shaded areas represent ±4 SD around the mean. Insets provide a zoom-in of vesicles with D < 15 μm. Successful encapsulation defines a condition in which the fluorescence within a GUV exceeds the average background intensity by four standard deviations (SD). For ρ_I = 1040 kg/m³, the background fluorescence intensity is 0.01 ± 0.01 (mean ± 4SD) and the encapsulation efficiency is 94.8% (N_{GUVs} = 96). Similarly, for ρ_I = 1060 kg/m³, the background fluorescence intensity is 0.02 ± 0.01 (mean ± 4SD) and the encapsulation efficiency is 94.5% (N_{GUVs} = 274). At the highest density ρ_I = 1110 kg/m³, the background intensity is 0.008 ± 0.008 (mean ± 4SD), and the encapsulation efficiency is 97.5% (N_{GUVs} = 611). Conditions: experiments are performed in the absence of salt (i.e., 0 mM KCl). For clarity, the data shown is from a single representative experiment.

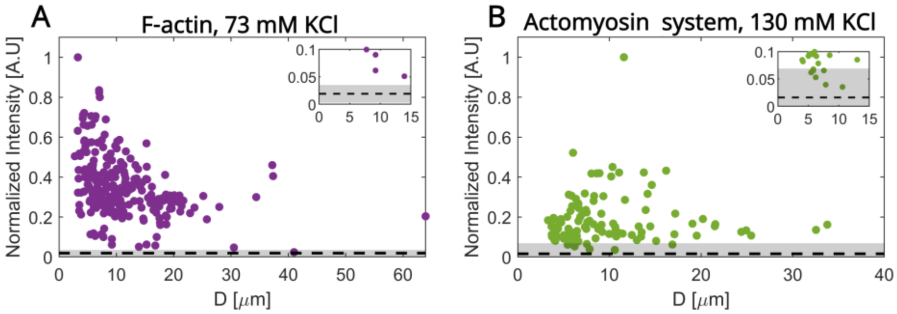

Figure S2: Normalized fluorescence intensity of GUVs as a function of vesicle diameter (*D*) for different cytoskeletal actin networks and salinity. Each panel shows the normalized fluorescence intensity (A.U.) of individual GUVs plotted against their diameter (*D*) for different encapsulated systems: A) includes a solution with pre-formed actin filaments (F-actin) and 73mM KCl and B) includes a solution of actin monomers, fascin, and myosin II motors that polymerizes in the presence of 130 mM KCl into an actomyosin network. Fluorescence intensities were normalized to the maximum value measured within each experiment. The dashed lines indicate the mean background intensity, and the shaded areas represent ±4 SD around the mean. Insets provide a zoom-in of vesicles with D < 15 µm. Successful encapsulation defines a condition in which the fluorescence within a GUV exceeds the average background intensity by four standard deviations (SD). Conditions: (A) the background fluorescence intensity is 0.02 ± 0.02 (mean ± 4SD) and the encapsulation efficiency is 99.6% (N_{GUVs} = 253) and (B) the background fluorescence intensity is 0.02 ± 0.05 (mean ± 4SD) and the encapsulation efficiency is 92.7% (N_{GUVs} = 109). For clarity, the data shown is from a single representative experiment.
